# Real-world chronic recordings from implantable adaptive deep brain stimulation systems for Parkinson’s disease motor state classification

**DOI:** 10.3389/fbinf.2026.1820165

**Published:** 2026-06-15

**Authors:** Hanan Awad Hassan Ali, Matteo Guidetti, Laura Caffi, Mattia Arlotti, Lorenzo Rossi, Chiara Palmisano, Alberto Priori, Francesco Brun, Ioannis Ugo Isaias, Sara Marceglia

**Affiliations:** 1 Dipartimento di Matematica, Informatica e Geoscienze, Università degli Studi di Trieste, Trieste, Italy; 2 Faculty of Computers and Informatics, Suez Canal University, Ismailia, Egypt; 3 Dipartimento di Scienze della Salute, Aldo Ravelli Research Center for Neurotechnology and Experimental Neurotherapeutics, Università degli Studi di Milano, Milan, Italy; 4 University Hospital of Würzburg and Julius Maximilian University of Würzburg, Würzburg, Germany; 5 The BioRobotics Institute, Sant’Anna School of Advanced Studies, Pisa, Italy; 6 Newronika SpA, Milan, Italy; 7 Fondazione Pezzoli per la Malattia di Parkinson, Milan, Italy; 8 Department of Engineering and Architecture, University of Trieste, Trieste, Italy; 9 Trieste Division, National Institute for Nuclear Physics (INFN), Trieste, Italy; 10 Parkinson Institute of Milan, ASST G. Pini-CTO, Milano, Italy

**Keywords:** deep brain stimulation, deep learning, Parkinson’s disease, subthalamic local field potentials, wearable sensors

## Abstract

**Introduction:**

Precise identification of motor states in Parkinson’s disease (PD) is critical for adaptive deep brain stimulation (aDBS) therapies. Recent developments in implantable neurostimulators now support continuous neural recordings, enabling long-term monitoring of PD-related neural dynamics under real-world conditions. However, the real potential of such deep brain continuous recordings in chronic home-based conditions has not been explored yet. In this work, we introduce a multimodal classification framework that integrates subthalamic nucleus local field potentials (STN-LFPs), wearable sensors, and patient-reported diaries to distinguish between three key motor states: *ON*, *OFF*, and *SLEEP*.

**Methods:**

The framework was initially validated on data collected from three patients over an average of 30 days (totaling 2,136 h of LFP recordings), and then applied to a larger dataset from thirteen patients recorded over an average of 14 days (totaling 4,440 h of LFP recordings). Feature extraction was performed in the time, frequency and time-frequency domains, after applying principal component analysis (PCA) to decrease dimensionality, which preserved 95% of the variance of the data and reduced computational complexity.

**Results:**

The multilayer perceptron (MLP) classifier using time–frequency domain features achieved the highest F1-score among the models tested. For the three validation patients, the MLP reached F1-scores of 81.1% with wearable sensor data and 94.3% with diary-derived data. When evaluated across all thirteen patients, it maintained a strong F1-score of 93.1% using diary time–frequency features.

**Discussion:**

These results demonstrate that chronic LFP recordings available 24/7 from implantable aDBS devices enable robust motor state classification, thus supporting the personalization and optimization of aDBS systems for real-life use.

## 1 Introduction

Parkinson’s disease (PD) is a prevalent neurodegenerative condition, affecting approximately 1%–3% of people aged over 60 (Pringsheim et al., 2014; McGregor and Nelson, 2019). It presents primarily with motor symptoms such as bradykinesia, tremor, rigidity, and postural instability (Jankovic et al., 1990; Olanow, 1990; Hebb et al., 2012; Powell et al., 2011), which differ among individuals and fluctuate over time.

Deep brain stimulation (DBS) is a neurosurgical therapy widely used for the treatment of advanced PD. By the chronic delivery of high-frequency (100–180 Hz) electrical stimulation through implanted electrodes targeting subcortical brain structures, most commonly the subthalamic nucleus (STN) or the globus pallidus internus (GPi) DBS can significantly alleviate motor symptoms (Perlmutter and Mink, 2006; Little and Brown, 2012). Traditional continuous DBS (cDBS) applies constant stimulation throughout the day, regardless of the patient’s clinical state and is often associated to side effects such as speech, gait, or sleep impairments (Perlmutter and Mink, 2006; Meidahl et al., 2017; Krack et al., 2017). In contrast, new adaptive DBS (aDBS) approaches aim to deliver stimulation only when needed, however requiring reliable biomarkers and decoding algorithms to detect the patient’s state (Priori et al., 2013; Cagnan et al., 2019; Marceglia et al., 2021; Guidetti et al., 2021). Accurate decoding of patient’s states is therefore essential to support real-time adjustments of closed-loop deep brain stimulation.

Local field potentials (LFPs) are electrophysiological signals that reflect the compound activity of neuronal populations within a specific brain region. In PD, LFPs can be continuously recorded from DBS electrodes implanted in subcortical targets (STN, GPi). In these patients, early studies primarily focused on LFPs oscillatory activity in the beta frequency range, which is closely associated with impaired motor function and modulated by movement and dopaminergic therapy (Kuhn et al., 2006; Brown, 2003). Because beta activity is associated with motor symptoms and respond to DBS and pharmacological therapy, LFPs represent a practical and informative signal for characterizing motor states in patients with PD (Golshan et al., 2018; Little and Brown, 2012; Friston et al., 2015; Williams et al., 2002; Marceglia et al., 2021; Potel et al., 2022; Marceglia et al., 2011).

Early studies primarily focused on oscillatory activity in the beta frequency range, which is closely associated with impaired motor function and is modulated by movement and dopaminergic therapy (Kühn et al., 2006; Brown, 2003; Priori et al., 2004), thus motivating the use of beta STN-LFPs for decoding motor and behavioral states in PD. However, other frequency bands, such as gamma (Little and Brown, 2012; Lofredi et al., 2018), and other approaches based on time features, such as beta-bursts (Piña-Fuentes et al., 2019), were also proposed and applied (Swann et al., 2018; Oehrn et al., 2024; Wilkins et al., 2025).

To allow adaptive approaches optimization as well as improved basal ganglia neurophysiology understanding, several approaches have everaged STN-LFPs using engineered features such as wavelet entropy, Hjorth parameters, phase-amplitude coupling (PAC), and burst analysis, combined with machine learning (ML) models including support vector machines, XGBoost, and Kalman filters (Yao et al., 2020; Houston et al., 2019). For example, Loukas and Brown demonstrated that rhythmic oscillations in STN-LFPs can be used to detect self-initiated hand movements (Loukas and Brown, 2004), while Golshan et al. introduced a multiple kernel learning approach to classify behavioral tasks based on STN-LFP features (Golshan et al., 2016). More recent studies have moved toward personalized motor state decoding. For example, Peterson et al. (2021) combined electrocorticography (ECoG) and STN-LFP recordings using spatio-spectral features, demonstrating the benefit of integrating spatial information. Similarly, dimensionality reduction techniques such as principal component analysis (PCA) and wavelet transforms have been used to improve classification performance in human behavior decoding tasks (Golshan et al., 2020; Niketeghad et al., 2014).

Despite these efforts, most existing studies remain limited by small sample sizes, short-term laboratory recordings, or reliance on a single signal modality, which may restrict their generalizability to real-life settings. With the availability of novel implantable pulse generators (IPGs) for DBS allowing aDBS delivery and LFP sensing during stimulation at home and over long time periods (Arlotti et al., 2021; Stanslaski et al., 2024), these limitations should be overcome.

First attempts to use long-term recordings from implanted DBS systems showed that available signals are reliable for supporting programming, paving the way towards electrophysiological-informed automatic programming approaches (Falciglia et al., 2025). In addition, sensing-enabled IPGs have enabled chronic wireless recording of subthalamic LFPs, showing the feasibility of long-term neural monitoring beyond short laboratory sessions (Gilron et al., 2021; Ramesh et al., 2026). However, despite these advances, the use of signals recorded from sensing-enabled IPGs in chronic at-home conditions for advanced real-time motor-state prediction remains largely unexplored.

For this reason, in this work we aimed to demonstrate the feasibility of using chronically-recorded STN LFPs to extract multimodal features enabling motor state decoding across different patients, leveraging a large dataset of more than 4,400 h of LFPs recorded 24/7 from 13 patients for 1 month, and integrating wearable sensors, and patient-reported diaries.

## 2 Materials and methods

**FIGURE 1:**
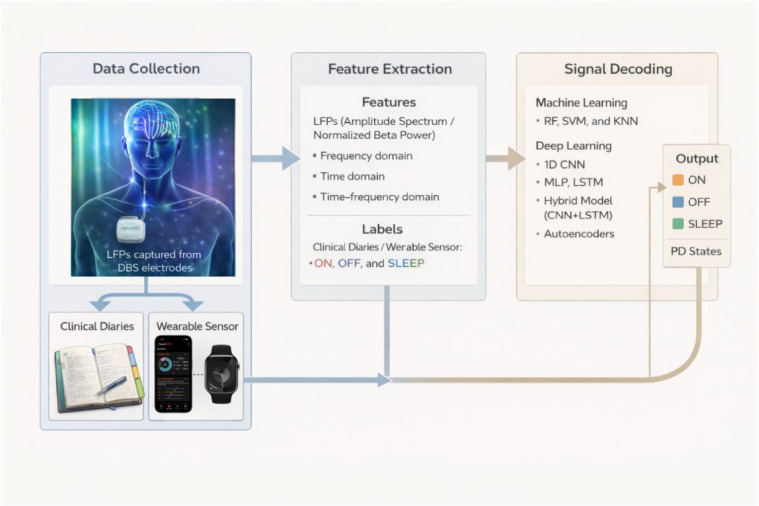
System Overview. The proposed framework integrates data from DBS electrodes, wearable sensors, and clinical diaries. Features are extracted across frequency, time, and time-frequency domains from LFPs, while motor state labels are derived from clinical diaries and wearable sensor data. Machine and deep learning models are used to classify the motor states (ON, OFF, and SLEEP) based on the extracted features.

This section describes the multimodal framework used to decode motor states in PD, integrating neural signals, wearable sensor data, and clinical diaries. The overall processing pipeline, from data acquisition to motor state classification, is summarized in Figure 1. The following subsections detail the experimental protocol, patient state recording, feature extraction, and preprocessing steps.

### 2.1 Experimental protocol

#### 2.1.1 Patients

Thirteen patients with PD (2 women and 11 men; age range 45–74 years) were included in this study (Table 1). All patients were implanted bilaterally in the STN with the AlphaDBSipg IPG (Newronika SpA, Italy), a bidirectional neurostimulation system capable of delivering DBS while chronically recording LFPs (Arlotti et al., 2021).

**TABLE 1:** Demographic and clinical characteristics.

Characteristic	BR group (N = 7)	DN group (N = 6)
Age (years)	57.7 (8.3)	61.3 (6.2)
BMI (kg/m^2^)	26.3 (2.7)	28.3 (8.6)
Male, N (%)	6 (85.7%)	5 (83.3%)
Female, N (%)	1 (14.3%)	1 (16.7%)
Disease duration (years)	16.6 (5.4)	14.7 (3.4)
Quadripolar leads	7 (100%)	3 (50%)
Directional leads	0 (0%)	3 (50%)

Values are mean (SD) or N (%). BR: IPG replacement group; DN: New DBS implant group. BMI: Body Mass Index.

Six patients were implanted with directional Abbott 6,172 electrodes (Abbott Inc., United States), and seven patients with Medtronic 3,389 electrodes (Medtronic Inc., United States). Each patient followed a structured monitoring protocol that included periods of cDBS and aDBS, each lasting approximately 2 weeks, as defined in the clinical study protocol (Marceglia et al., 2022).

During cDBS, stimulation was delivered continuously using clinically optimized fixed parameters (see (Hirschmann et al., 2013) for details). During aDBS, stimulation amplitude was automatically adjusted once per minute using a linear proportional control algorithm driven by beta-band activity recorded from the STN, as previously described in detail (Caffi et al., 2024). The control signal (i.e., the beta amplitude) was computed as a smoothed estimate of the average LFP amplitude within a patient-specific beta frequency range, normalized to the total spectral amplitude between 5 and 34 Hz (Arlotti et al., 2021). A single electrode contact, referred to as the driving channel, was selected for control based on signal stability and clinical relevance (Table 2).

**TABLE 2:** Programming details of DBS patients.

Patient N	Side	Pulse (µs)	Freq (Hz)	cDBS (mA)	aDBS min (mA)	aDBS max (mA)	Modulation	Max–cDBS	Biomarker (Hz)	Driving channel
Patient 1	Left	60	130	2.3	2.1	2.5	0.4	+0.2	12–20	Right
	Right	60	130	2.8	2.6	3.0	0.4	+0.2	12–20	Right
Patient 2	Left	70	130	2.4	2.6	3.9	1.3	+1.5	11–16	Left
	Right	70	130	2.5	2.6	3.0	0.4	+0.5	11–16	Left
Patient 3	Left	60	130	2.5	2.4	3.4	1.0	+0.9	11–16	Right
	Right	60	130	2.5	2.4	3.2	0.8	+0.7	11–16	Right
Patient 4	Left	60	70	4.0	3.5	4.5	1.0	+0.5	12–19	Right
	Right	60	70	3.5	3.0	4.0	1.0	+0.5	12–19	Right
Patient 5	Left	60	130	2.2	1.8	2.8	1.0	+0.6	12–18	Right
	Right	60	130	2.1	1.8	2.8	1.0	+0.7	12–18	Right
Patient 6	Left	60	130	3.0	2.0	3.3	1.3	+0.3	12–20	Right
	Right	60	130	3.1	1.0	3.1	2.1	0.0	12–20	Right
Patient 7	Left	60	80	4.0	2.5	4.5	2.0	+0.5	11–17	Left
	Right	60	80	4.0	3.0	4.5	1.5	+0.5	11–17	Left
Patient 8	Left	60	130	2.4	2.0	2.9	0.9	+0.5	11–20	Left
	Right	60	130	2.3	2.0	3.5	1.5	+1.2	11–20	Left
Patient 9	Left	60	130	2.0	1.5	2.6	1.1	+0.6	9–16	Right
	Right	60	130	2.2	1.5	2.6	1.1	+0.4	9–16	Right
Patient 10	Left	60	130	2.3	1.5	3.0	1.5	+0.7	12–20	Left
	Right	60	130	1.8	1.2	1.8	0.6	0.0	12–20	Left
Patient 11	Left	60	130	1.0	1.0	2.5	1.5	+1.5	12–18	Left
	Right	60	130	1.0	0.8	2.0	1.2	+1.0	12–18	Left
Patient 12	Left	60	130	2.2	1.0	2.7	1.7	+0.5	12–18	Right
	Right	60	130	1.8	0.8	2.5	1.7	+0.7	12–18	Right
Patient 13	Left	60	130	2.2	2.0	2.5	0.5	+0.3	12–17	Left
	Right	60	130	2.2	2.0	2.5	0.5	+0.3	12–17	Left

Values are reported per hemisphere. Abbreviations: Min and Max denote minimum and maximum stimulation amplitudes; aDBS, adaptive deep brain stimulation; cDBS, conventional deep brain stimulation.

#### 2.1.2 LFP recordings

The AlphaDBSipg stores the beta amplitude every minute (per each channel) and one full amplitude spectrum (5–34 Hz range) every 10 min per channel with 1 Hz resolution (Arlotti et al., 2021). The stored signals are then automatically downloaded to the patient controller (AlphaDBSpat) during daily recharging sessions, ensuring no data loss across long time windows. Every minute, the beta amplitude is stored both as “raw” beta amplitude (representing the integral of the amplitude spectrum within the within the patient-specific beta frequency range), and as “normalized” beta amplitude, obtained by dividing the raw value by the total spectral amplitude between 5 and 34 Hz. These neural recordings formed the basis for feature extraction and motor state decoding.

#### 2.1.3 Patient state recording

Patient motor states were recorded using a combination of clinical diaries and wearable sensors. While all patients collected the clinical diary (Hauser PD diary (Hirschmann et al., 2016)), only three patients had both wearable sensors and diaries.

Clinical diaries were completed every 30 min and included annotations of sleep periods (SLEEP) and motor state, distinguishing between OFF and ON states with different levels of dyskinesia (no dyskinesias, mild dyskinesias, severe dyskinesias). To match the resolution of LFP recordings, each diary entry was expanded to 1-min resolution by assigning the reported state to all minutes within the corresponding 30-min interval.

Wearable sensor data were collected at 15–minute resolution using a wrist-worn device that provided objective measurements of motor activity, including automated detection of bradykinesia, dyskinesia, and tremor, as well as inferred ON/OFF motor states. Motor–state labels were assigned using rule–based criteria: intervals marked as OFF or showing significant bradykinesia or tremor were labeled as OFF, whereas intervals marked as ON or showing dyskinesia were labeled as ON. Periods flagged as invalid were excluded, and non–wear periods were grouped under the SLEEP category. Each 15–minute sensor interval was then expanded to 1-min resolution to align with neural recordings.

For the purpose of state recognition, all ON subcategories were grouped into a single ON state, thus collapsing the entries to three categories, namely, *OFF* (patients experiencing motor symptoms and impaired movements), *ON* (patients feeling able to move), and SLEEP. Only time points with overlapping data from all sources were retained, resulting in 24–30 usable diary days and 25–37 valid wearable sensor days for three patients. Additionally, between 1 and 30 days of diary data were available for all thirteen patients.

Usable diary days and wearable-sensor days did not necessarily coincide because the two modalities were collected and processed independently. After temporal alignment with LFP recordings, time windows were excluded when any signal was missing or invalid, such as incomplete diary entries, missing or corrupted sensor recordings, or missing LFP data. Therefore, the number of usable days refers to days with sufficient valid data for each data source separately, and does not necessarily correspond to the same calendar days across data sources. In addition, because clinical diaries reflect patient-reported motor perception whereas wearable sensors infer motor state from movement features, the two labeling approaches may produce different labels in some time windows.

### 2.2 Feature extraction

To extract a diverse and informative set of biomarkers, features were derived across frequency, time, and time-frequency domains. These features were designed to capture both stable and transient aspects of neural activity, offering a richer representation for classification. All extracted features were organized into non-overlapping 10-min windows, so that each sample in the dataset corresponds to a single 10-min interval used for model training and evaluation. The left and right STN recordings were categorized into driving and non-driving channels, according to the ongoing aDBS programming.

#### 2.2.1 Frequency domain features

**FIGURE 2:**
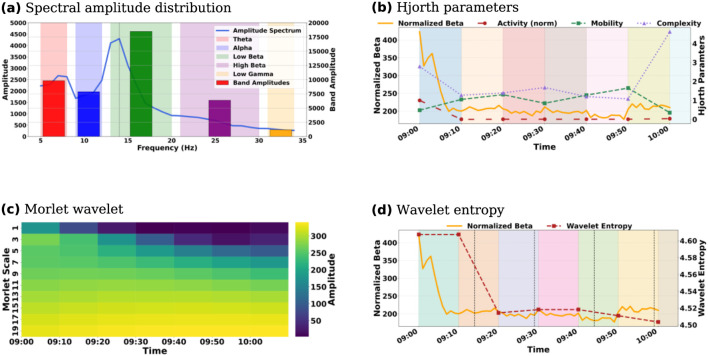
Representative feature representations extracted from LFPs. **(a)** Spectral amplitude distribution across defined frequency bands, including theta, alpha, low beta, high beta, and low gamma. **(b)** One-hour overview of normalized beta activity alongside Hjorth parameters, where shaded regions indicate the 10-min intervals used for feature extraction. **(c)** Time–frequency representation of beta activity generated using the Morlet continuous wavelet transform, showing the first 10 wavelet scales with color intensity indicating amplitude variations over time. **(d)** One-hour overview of normalized beta activity alongside wavelet entropy, where each entropy value is computed over distinct 15-min intervals and applied uniformly across its corresponding time segment.

The frequency-based features were obtained by analyzing the amplitude spectrum of LFP signals between 5 and 34 Hz for each channel. To capture specific brain activity patterns linked to PD, we calculated the total amplitude within commonly studied frequency bands: theta (5–8 Hz), alpha (9–12 Hz), low beta (13–20 Hz), high beta (21–30 Hz), and low gamma (31–34 Hz) bands for each STN channel. These values were computed separately for both channels by summing the amplitudes within each band, as shown in Figure 2a.

#### 2.2.2 Time domain features

To characterize the temporal structure of neural activity, we computed a variety of statistical and dynamic metrics using LFP data. These features were derived from spectral amplitude data and normalized beta amplitude.

- Statistical Metrics: We extracted basic statistical measures from the amplitude spectrum such as the mean, standard deviation (STD), root mean square (RMS), peak-to-peak (PTP) amplitude, energy, and entropy. These parameters summarize the signal’s average behavior, variability, and amplitude distribution over time for each LFP channel.
•Hjorth Parameters: To capture the dynamic characteristics of beta activity, we computed the Hjorth descriptors—*Activity*, *Mobility*, and *Complexity* Hjorth (1970) (Equations 1–3), from the normalized beta signal using non-overlapping 10-min windows, as illustrated in Figure 2b.
  ○ Activity reflects the amount of variation in the signal, representing the magnitude of amplitude fluctuations:
$$A_{x}=\frac{1}{N}\overset{N}{\underset{n=1}{\sum }}{\left[x\left[n\right]-\mu_{x}\right]}^{2}$$ (1)
    where x[n] denotes the discrete signal, $\mu_{x}$ represents the average value of the signal, and N indicates the number of data points.
  ○ Mobility describes the main frequency characteristics by evaluating how much the signal’s rate of change varies compared to the signal itself:
$$M_{x}=\sqrt{\frac{\text{var}\left(\dot{x}\left[n\right]\right)}{\text{var}\left(x\left[n\right]\right)}}$$ (2)
    where $\dot{x}\left[n\right]$ is the first derivative of the signal.
  ○ Complexity reflects how frequency patterns change over time, calculated as the ratio of mobility between the first derivative and the original signal:
$$C_{x}=\frac{M_{\dot{x}}}{M_{x}}$$ (3)
    where $M_{\dot{x}}$ denotes the mobility calculated from the first derivative of the signal.

#### 2.2.3 Time–frequency domain features

Time–frequency analysis of LFP signals, especially within the beta frequency range, has proven effective for distinguishing motor behaviors and capturing dynamic changes in neural activity (Golshan et al., 2016; 2017; 2018; Niketeghad et al., 2017). Prior studies have shown that features derived from wavelet transforms can reflect fluctuations in motor symptoms and provide insight into responses to medication or stimulation (Hebb et al., 2012; Hanrahan et al., 2016). In contrast to classical approaches that apply time–frequency analysis directly to raw LFP time–series, we analyzed the temporal evolution of normalized beta activity. To capture both spectral and temporal variations in this biomarker, we employed the Continuous Wavelet Transform (CWT) using Morlet wavelets (Equations 4–5) applied to the normalized beta activity. This approach characterizes the modulation dynamics of beta activity over time.

- Morlet CWT: We applied the Morlet wavelet transform from each channel to multiple scales. The CWT enables simultaneous time and frequency analysis by applying scaled versions of the wavelet function to the input signal. For each recording, we computed the wavelet coefficients using scales ranging from 1 to 99, with a step of 2, to capture time–frequency characteristics across different frequency components of the signal. The magnitude of these coefficients was used to form a multiscale representation of the signal:

$$W_{x}\left(a,b\right)=\int_{-\infty }^{\infty }x\left(t\right)\psi^{*}\left(\frac{t-b}{a}\right)dt$$ (4)

here, x(t) represents the signal being analyzed, ψ is the Morlet wavelet, a controls the scale, b shifts the wavelet in time, and the symbol ∗ indicates complex conjugation. The resulting CWT coefficients were stored as additional features at each scale. As shown in Figure 2c.

- Wavelet Entropy: To quantify the degree of disorder or complexity within the wavelet-transformed signal, we computed wavelet entropy Rosso et al. (2001). This metric reflects how energy is distributed across wavelet scales and serves as an indicator of signal irregularity.

Wavelet entropy was computed for each non-overlapping 15-min window, and the same value was assigned to all minutes within that window, as shown in Figure 2d. Specifically, the signal was first transformed using the Morlet CWT, then the relative energy at each scale was obtained, and finally, Shannon entropy was calculated based on the normalized energy distribution across scales. The use of wavelet entropy enables robust characterization of time-varying complexity, offering a complementary perspective to static spectral or time-domain measures. The wavelet entropy $E_{w}$ is given by:

$$E_{w}=-\overset{N}{\underset{i=1}{\sum }}p_{i}\log\left(p_{i}\right)$$ (5)

where $p_{i}$ is the normalized energy at the i -th wavelet scale, and N represents the total number of wavelet scales. This feature was assigned uniformly to all samples within each time window, ensuring temporal alignment with the underlying data resolution.

### 2.3 Feature relevance analysis

**FIGURE 3:**
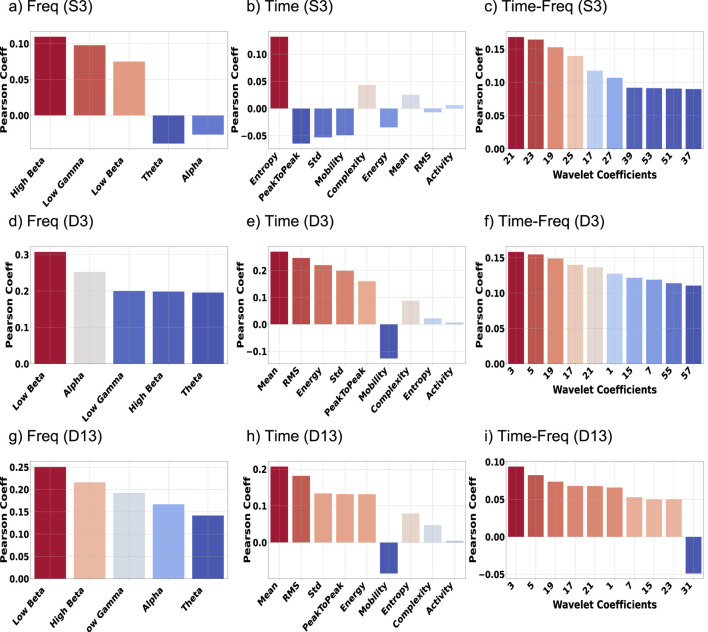
Pearson correlation analysis between extracted features from the driving channel and PD motor states. **(a–c)** Wearable sensor data of 3 patients (S3): frequency, time, and time–frequency domains. **(d–f)** Diary data of 3 patients (D3): frequency, time, and time–frequency domains. **(g–i)** Diary data of 13 patients (D13): frequency, time, and time–frequency domains.

To determine which features were most indicative of PD motor states, we computed Pearson correlation coefficients between extracted neural features and motor state labels across both wearable sensor and clinical diary datasets, as shown in Figure 3. Statistical significance was corrected for multiple comparisons using the Benjamini–Hochberg false discovery rate (FDR) procedure.

In the subset of three patients with both sensor and diary data, several features showed statistically significant associations with motor states after FDR correction. For sensor data (Figures 3a–c), effect sizes were generally modest. In the driving hemisphere, high beta, low beta, and low gamma band amplitude, together with time-domain metrics such as entropy and peak-to-peak amplitude, and early Morlet wavelet coefficients, were among the most correlated features. In contrast, in the non-driving hemisphere, fewer time and frequency domain features remained significant after FDR correction, although several wavelet coefficients still showed statistically significant but moderate correlations. When analyzing diary data in the same subset (Figures 3d–f), low beta and alpha amplitude, as well as time-domain features such as mean and RMS, were among the most relevant features in the driving hemisphere. The non-driving hemisphere generally exhibited smaller correlation values across feature domains.

Extending this analysis to the thirteen patients with diary data only (Figures 3g–i), low beta, high beta, and low gamma amplitude emerged among the most correlated features with motor states in the driving hemisphere. Time-domain features such as mean, RMS, and STD were also statistically significant after FDR correction. Early-scale wavelet features showed smaller yet significant correlations, consistently ranking among the most informative time–frequency features in the driving hemisphere, whereas their relevance was reduced in the non-driving hemisphere.

In addition, confidence intervals for Pearson correlation coefficients were estimated using bootstrap resampling for features extracted from all patients in the driving channel. The results showed narrow confidence intervals, confirming the stability of the observed associations. The strongest correlations were observed in the beta frequency band (e.g., low beta: r≈0.25 , 95% CI [0.24–0.26]).

To further capture potential nonlinear relationships, mutual information was computed between neural features and motor states for the same dataset. The results were consistent with the correlation analysis, highlighting beta-band features and wavelet entropy as among the most informative features, although effect sizes remained modest (maximum MI ≈0.15 ).

Although these features were statistically significant, their effect sizes were relatively small (|r|<0.3) , indicating that individual features alone are not sufficient to reliably distinguish motor states.

### 2.4 Pre-processing

#### 2.4.1 Dataset labeling

Each feature was assigned one of the three motor states: *ON*, *OFF*, or *SLEEP*, based on annotations derived from clinical diaries and wearable sensor recordings (re-sampled to 1 min; see Section 2.1.2).

For the three patients with both diaries and sensors, diary-based annotations yielded a total of 8,707 labeled samples: 5,051 labeled as *ON*, 815 as *OFF*, and 2,641 as *SLEEP*. In contrast, wearable sensor–based annotations for the same patients resulted in 10,063 labeled samples: 6,774 *ON*, 673 *OFF*, and 2,616 *SLEEP*.

When extended to all thirteen patients with diary annotations, this process yielded 20,723 labeled entries: 11,568 *ON*, 2,247 *OFF*, and 6,908 *SLEEP*.

Only time windows with neural recordings and valid motor-state labels from diaries or wearable sensors were retained for analysis. Invalid data periods were defined as segments containing missing values, signal artifacts, or incomplete recordings, and were excluded from the analysis, resulting in fewer labeled samples than the number of recorded days.

In addition to computing the overall agreement across the three motor states (ON, OFF, SLEEP), we performed a more detailed analysis by evaluating Cohen’s kappa for specific binary classifications. In particular, agreement was assessed for ON versus OFF states (excluding SLEEP samples) and for SLEEP versus non-SLEEP states (combining ON and OFF). These analyses were performed on temporally aligned data using the same 10-min windows employed for feature extraction.

#### 2.4.2 Features alignment

Features and labels were originally available at different temporal resolutions. Spectral features derived from the stored STN amplitude spectrum were computed at a 10-min rate, matching the device saving interval. Beta-related features (normalized beta) were available at 1-min resolution; however, Hjorth parameters and time–frequency features were extracted from the normalized beta signal using non-overlapping 10-min windows, yielding one feature vector per 10-min segment. Wavelet entropy was computed over non-overlapping 15-min windows and assigned uniformly to all samples within each window before temporal alignment.

To align all data sources, diary and sensor labels were first converted to 1-min resolution by assigning the reported state to each minute within its interval. Then, for each 10-min neural recording window, the corresponding label was obtained from the aligned 1-min labels. In this way, each sample in the dataset represents one 10-min neural segment paired with the motor-state label valid during that same time period. This procedure ensured temporal alignment between features and motor-state labels for model training and evaluation.

Agreement between clinical diary–based and wearable sensor–derived motor state labels was evaluated using Cohen’s kappa coefficient on temporally aligned data from the subset of three patients with both modalities.

#### 2.4.3 Dimensionality reduction

Dimensionality reduction aims to simplify the feature space used for detecting patient states by eliminating redundant or less informative variables. Using too many features can lead to overfitting, which reduces the model’s ability to generalize well to unseen data. To address the high dimensionality of the extracted features and enhance computational efficiency, PCA was applied. Features were first standardized using *z-score* normalization, followed by decomposition of the covariance matrix to extract eigenvectors and eigenvalues. Components were selected based on their contribution to total variance, retaining only those necessary to explain 95% of the cumulative variance, a commonly adopted threshold that balances dimensionality reduction with preservation of informative signal structure. This step helped preserve the most informative characteristics while minimizing noise and redundancy in the dataset.

This resulted in retaining 5 components for frequency features, 9 for time features, and 20 for time–frequency features. For combined feature sets, 9 components were retained for frequency and time features, 23 for frequency and time–frequency features, and 27 components for both time and time–frequency features as well as for the full feature set.

While PCA improves computational efficiency and reduces redundancy, it may limit the direct interpretability of individual features, as each principal component represents a combination of multiple neurophysiological variables.

## 3 Model definiton and evaluation

### 3.1 Machine learning models

We evaluated three classical machine learning algorithms: The Random Forest (RF), Support Vector Machine (SVM), and K-Nearest Neighbors (KNN), each trained on standardized feature sets and optimized via grid search. RF operates as an ensemble method that combines the predictions of multiple decision trees through majority voting. It is particularly effective on high-dimensional datasets and does not require extensive preprocessing. In our experiments, we tuned hyperparameters including the number of trees (n_estimators), tree depth (max_depth), and node splitting criteria (min_samples_split, min_samples_leaf).

The SVM is supervised learning algorithm that classify data by identifying a decision boundary known as a hyperplane that maximally separates different classes. We explored various kernel functions including linear, polynomial, radial basis function (RBF), and sigmoid, allowing the model to capture both linear and nonlinear relationships. We adjusted the regularization strength and the kernel setting to improve the model’s performance.

The KNN is a non-parametric technique that assigns labels to new data by identifying the most frequent class among the k nearest data points in the feature set. The effectiveness of the model depends significantly on the underlying data distribution and the selected distance measure used to identify nearby points. We adjusted the value of k to determine the optimal number of neighbors, various distance metrics were tested, including Euclidean, Manhattan, and Minkowski, along with an assessment of weighting methods such as uniform and distance-based.

Random oversampling was applied only to the training data after the train–test split to address class imbalance and avoid data leakage. Standardization was applied before training to ensure fair distance computation. Model hyperparameters for each classifier were optimized using a 5-fold cross-validation strategy and the performance of the model was assessed using an independent test set.

### 3.2 Deep learning models

**FIGURE 4:**
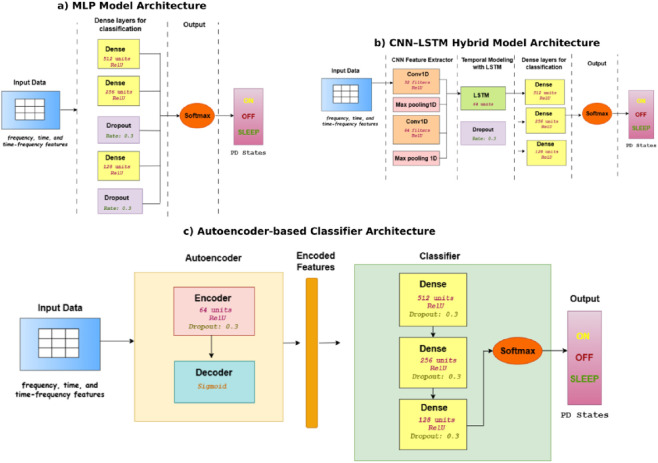
Model architectures evaluated for Parkinson’s disease motor state classification. **(a)** Multilayer perceptron (MLP) architecture, where the input feature matrix composed of frequency-, time-, and time–frequency-domain features is processed through three fully connected dense layers with ReLU activation and dropout regularization, followed by a softmax layer for classification. **(b)** CNN–LSTM hybrid architecture, in which one-dimensional convolutional layers extract local feature representations that are passed to an LSTM unit for temporal modeling, followed by dense layers and a softmax output. **(c)** Autoencoder-based classifier architecture, where an encoder compresses the input features into a latent representation that is subsequently used by a dense classifier to predict Parkinson’s disease motor states (ON, OFF, and SLEEP).

All deep learning models were trained for 200 epochs. The selected architectures and hyperparameters were based on commonly used configurations in similar studies, balancing model complexity and generalization performance. The following architectures were explored. The multilayer perceptron (MLP) model employed three sequential dense layers with 512, 256, and 128 neurons, each activated by Rectified Linear Unit (ReLU) and regularized with a dropout rate of 0.3. A final softmax layer was used for multi-class classification, as shown in Figure 4a.

The one-dimensional convolutional neural networks (1D-CNN) architecture utilized two sequential convolutional layers with 32 and 64 filters, each employing ReLU activation, followed by max pooling and dropout for regularization. The resulting features were flattened and forwarded through three dense layers with 512, 256, and 128 neurons, respectively. A softmax layer at the end produced the class probabilities for prediction.

Long Short-Term Memory (LSTM) processed sequential input data through a single LSTM layer with 64 units, followed by three dense layers (512, 256, 128 units) with ReLU activations and dropout. The output was generated using a softmax classifier.

The CNN-LSTM architecture began with two 1D convolutional layers applying 32 and 64 filters to capture spatial characteristics, followed by max pooling to reduce dimensionality. These features were then forwarded to an LSTM layer to model sequential dependencies. The temporal features were refined using three fully connected layers consisting of 512, 256, and 128 neurons, and classification was performed via a softmax activation layer, as shown in Figure 4b.

The autoencoder consisted of an encoder that compressed the input feature vector into a 64-dimensional latent representation and a decoder that reconstructed the original input from this representation. The decoder output corresponds to reconstructed input data and was used only during autoencoder training to learn an informative latent space. After training, the encoder was retained and the latent features were extracted and used as input to a separate classifier. This classifier, composed of three sequential dense layers (512, 256, and 128 units with ReLU activation) followed by a softmax output layer, was trained independently to predict motor states. The full architecture is shown in Figure 4c.

Model performance was monitored on validation data during training, and learning curves were analyzed to ensure stable convergence and absence of significant overfitting. Although a fixed number of training epochs was used, validation curves indicated stable convergence, suggesting that the selected training duration was appropriate for the dataset.

### 3.3 Evaluation metrics

The dataset was divided into training and testing sets, with 80% of the data used for training and 20% for testing, ensuring that there was no overlap in time windows. To further assess how well the model performs across different recording days, we also used a leave-one-day-out approach. In this setup, data from one recording day per patient was used only for testing and was not included in the training data.

**FIGURE 5:**
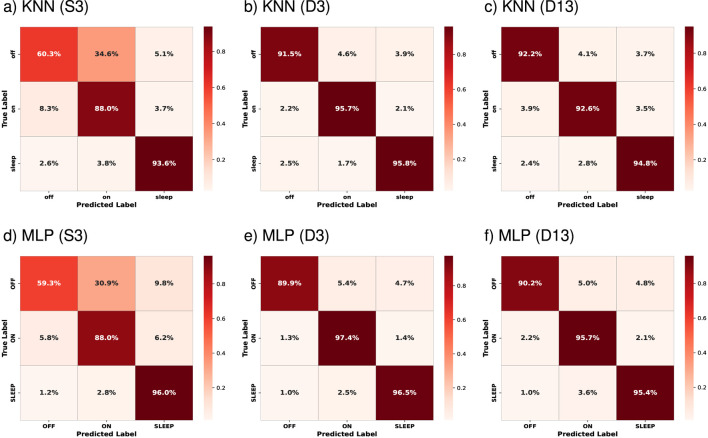
Confusion matrices for KNN and MLP classifiers across datasets. **(a–c)** KNN results for S3, D3, and D13, respectively. **(d–f)** MLP results for S3, D3, and D13, respectively. S3: wearable sensor data (3 patients); D3: diary data (3 patients); D13: diary data (13 patients).

**FIGURE 6 F6:**
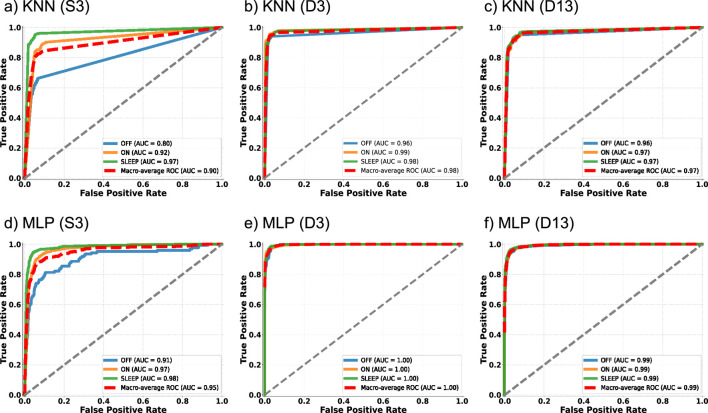
ROC curves for KNN and MLP classifiers across datasets. **(a–c)** KNN results for S3, D3, and D13, respectively. **(d–f)** MLP results for S3, D3, and D13, respectively. S3: wearable sensor data (3 patients); D3: diary data (3 patients); D13: diary data (13 patients).

**FIGURE 7:**
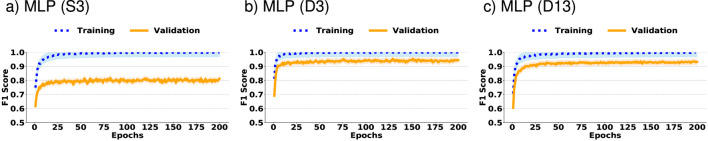
Training and validation F1-score curves for the MLP classifier. **(a)** S3 (wearable sensor data, 3 patients), **(b)** D3 (diary data, 3 patients), and **(c)** D13 (diary data, 13 patients).

**FIGURE 8:**
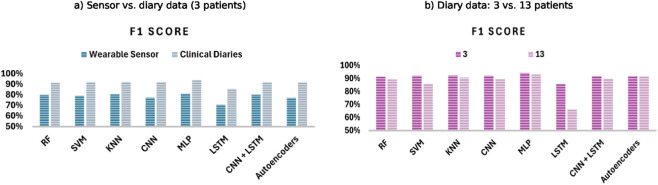
Comparison of F1-scores across datasets and patient groups. **(a)** Sensor versus diary data for three patients. **(b)** Diary data comparison between three and thirteen patients.

All classification results presented in Tables 3, 4 and Figures 5–8 were obtained using a random train–test split (80% training, 20% testing). A separate leave-one-day-out evaluation was conducted to assess model generalization to unseen recording days.

**TABLE 3:** F1-score (%) across feature sets for different classifiers and datasets. S3: Sensor data (3 patients); D3: Diary data (3 patients); D13: Diary data (13 patients).

Model	Dataset	Freq	Time	Time-freq	Freq + time	Time + time-freq	Freq + time-freq	All
RF	S3	58.6	51.0	79.9	56.3	72.0	77.1	72.7
	D3	66.3	57.1	91.4	60.4	88.5	91.6	87.2
	D13	69.4	64.8	89.2	66.8	84.5	88.2	84.7
SVM	S3	51.1	58.9	79.3	49.2	74.2	77.4	73.6
	D3	66.0	53.4	92.2	57.1	88.8	91.2	89.0
	D13	66.6	62.4	86.0	62.6	84.9	88.2	88.7
KNN	S3	51.9	47.3	**80.6**	47.7	74.6	78.7	74.3
	D3	60.8	48.6	92.4	55.2	89.9	**92.5**	89.8
	D13	64.9	55.8	**90.8**	57.3	86.9	90.7	86.3
CNN	S3	50.3	46.9	77.5	47.4	75.0	78.2	73.0
	D3	62.9	52.0	92.2	57.3	88.9	92.8	89.0
	D13	63.6	60.3	89.5	61.0	87.6	89.6	88.1
MLP	S3	53.9	53.8	**81.1**	51.2	75.8	78.5	74.4
	D3	64.7	55.3	**94.3**	59.7	91.4	92.9	90.7
	D13	66.9	63.9	**93.1**	64.8	90.6	92.7	90.7
LSTM	S3	51.9	47.6	70.7	45.5	58.9	70.7	63.2
	D3	61.7	49.7	85.8	53.7	76.2	84.5	75.9
	D13	64.0	59.4	66.2	60.8	75.3	81.0	73.4
CNN + LSTM	S3	55.0	46.7	80.1	48.5	69.2	76.5	71.0
	D3	61.6	51.3	91.9	57.8	86.8	92.6	85.0
	D13	67.3	60.6	89.7	60.9	83.8	89.1	83.7
Autoencoders	S3	53.7	50.5	77.3	51.2	75.4	80.0	76.2
	D3	63.6	52.9	91.7	58.6	90.3	92.4	90.0
	D13	65.5	61.5	91.5	63.0	87.9	91.0	87.7

Bold values denote the highest F1-score (%) achieved within each dataset, separately for machine learning and deep learning models.

**TABLE 4:** Classification performance of top models across datasets. S3: wearable sensor data (3 patients); D3: diary data (3 patients); D13: diary data (13 patients).

Model	Dataset	Class	Precision	Recall	F1-score	Support
KNN	S3	OFF	0.54	0.66	0.60	123
		ON	0.84	0.89	0.87	551
		SLEEP	0.98	0.94	0.96	1,329
	D3	OFF	0.80	0.92	0.85	153
		ON	0.98	0.96	0.97	1,024
		SLEEP	0.95	0.96	0.95	518
	D13	OFF	0.77	0.92	0.84	438
		ON	0.97	0.93	0.95	2,338
		SLEEP	0.93	0.95	0.94	1,361
MLP	S3	OFF	0.60	0.59	0.60	123
		ON	0.87	0.88	0.87	551
		SLEEP	0.97	0.96	0.96	1,329
	D3	OFF	0.88	0.90	0.89	149
		ON	0.98	0.97	0.98	1,024
		SLEEP	0.96	0.97	0.96	521
	D13	OFF	0.86	0.90	0.88	438
		ON	0.97	0.96	0.96	2,338
		SLEEP	0.95	0.95	0.95	1,361

Model performance was assessed using standard classification metrics: precision, recall, F1-score, ROC curves, and confusion matrices. Precision quantified the accuracy of positive predictions, while recall assessed the model’s ability to identify actual positive cases. The F1-score, which balances precision and recall, offered a comprehensive measure of model performance. All experiments were implemented in Python (v3.12.7), and key preprocessing, feature extraction, and model training steps were performed using standard scientific computing libraries. Detailed implementation can be made available upon reasonable request.

## 4 Results

This section presents the results of our multimodal classification framework for PD motor state recognition. We assess the discriminative power of extracted features and evaluate the performance of various machine and deep learning models across different data sources (wearable sensors and clinical diaries) and sample sizes (three vs. thirteen patients). Our objective was to identify the most effective feature combinations and classification approaches.

### 4.1 Classification performance

For the wearable sensor data from three patients, the MLP achieved the highest F1-score of 81.1% when using time-frequency domain features. The KNN classifier also performed well, reaching an F1-score of 80.6% with the same feature set. Other models, including RF, CNN, CNN-LSTM, and Autoencoders, achieved F1-scores above 77%, showing that all models were able to capture relevant patterns. Combining time-frequency and frequency features improved the performance of some models, with the KNN and Autoencoder reaching 78.7% and 80% F1-score, respectively.

For the clinical diary data from the same three patients, the MLP again outperformed other models, achieving an F1-score of 94.3% using time-frequency features. Other models, including RF, KNN, SVM, CNN, CNN-LSTM, and Autoencoders also performed remarkably, with F1-scores above 91% for time-frequency or combined feature sets. These results indicate that features extracted from LFPs and labeled using clinical diaries contain strong predictive temporal patterns, which are effectively captured by deep learning architectures.

When extended to the full cohort of thirteen patients, classification performance remained comparable to that observed in the three-patient subset, indicating good generalizability across subjects. The MLP maintained the highest performance, achieving a macro F1-score of 92.7% using time-frequency features, consistent with our previously reported performance of 93.1%. To assess the potential impact of cohort heterogeneity, we conducted subgroup analyses based on patient group and electrode type. Model performance was consistent across electrode types, with similar F1-scores observed for directional leads (92.7%) and quadripolar leads (92.9%) within the newly implanted patient group, indicating that electrode geometry does not significantly influence classification performance. In contrast, lower performance was observed in the (BR) IPG replacement group (82.2%) compared to the (DN) newly implanted group (86.8%), suggesting differences driven by patient-specific factors rather than methodological bias. Overall, these results show that our proposed approach is robust across electrode configurations while remaining sensitive to underlying physiological differences between patient groups. KNN and Autoencoders also maintained good performance, with F1-scores of 90.8% and 91.5%, respectively, confirming the generalizability of these approaches across a broader population. Combining frequency and time-frequency features slightly improved results for some models. For example, SVM improved from 86.0% using time–frequency features alone to 88.2% when both frequency and time–frequency features were combined. A minimal improvement was also observed for CNN (89.5%–89.6%), while LSTM showed a larger improvement (66.2%–81.0%). These classification results are summarized in Table 3.

To further investigate the impact of class imbalance on model performance, we evaluated several imbalance-handling strategies for the MLP model, including random oversampling, cost-sensitive learning, SMOTE, and ADASYN (Table 5).

**TABLE 5:** Comparison of imbalance-handling strategies using the MLP mode and time–frequency features on diary data from 13 patients.

Method	Class	Precision	Recall	F1-score	Support
No balancing	OFF	0.90	0.82	0.86	438
	ON	0.96	0.97	0.96	2,338
	SLEEP	0.94	0.95	0.95	1,361
Random oversampling	OFF	0.86	**0.90**	**0.88**	438
	ON	0.97	0.96	0.96	2,338
	SLEEP	0.95	0.95	0.95	1,361
Cost-sensitive learning	OFF	0.84	0.88	0.86	438
	ON	0.97	0.95	0.96	2,338
	SLEEP	0.94	0.96	0.95	1,361
SMOTE	OFF	**0.89**	0.85	0.87	438
	ON	0.97	0.96	0.96	2,338
	SLEEP	0.93	0.96	0.95	1,361
ADASYN	OFF	0.87	0.86	0.86	438
	ON	0.96	0.96	0.96	2,338
	SLEEP	0.95	0.95	0.95	1,361

Bold values indicate the highest Precision, Recall, and F1-score for the OFF class across all balancing methods.

All approaches improved the detection of the minority OFF class compared to the unbalanced baseline. Random oversampling achieved the highest macro F1-score (93.1%) and the highest OFF recall (0.90), but at the cost of reduced precision, indicating a higher rate of false positives. In contrast, the cost-sensitive approach provided a more balanced trade-off between precision and recall (OFF recall = 0.88) while maintaining stable overall performance (macro F1-score = 92.2%), without introducing duplicated or synthetic samples.

SMOTE and ADASYN yielded intermediate results (macro F1-scores of 92.7% and 92.4%, respectively) but did not consistently outperform the cost-sensitive approach.

To better illustrate model reliability, we present confusion matrices, classification reports and ROC curves for the best-performing machine learning models, along with training and validation F1-score curves for the top deep learning models, as shown in Figures 5, 6, [Fig F6] and Table 4. Across all datasets, the KNN classifier achieved the strongest machine learning results, while the MLP consistently provided the best deep learning performance. Using optimized parameters (metric=’manhattan’, weights=’distance’, and n_neighbors=3-4), KNN reached F1-scores of 80.6% (wearable sensor data, three patients, time-frequency features), 92.5% (diary data, three patients, combined time-frequency and frequency features), and 90.8% (diary data, thirteen patients, time-frequency features). The MLP outperformed KNN in all cases with F1-scores of 81.1%, 94.3%, and 93.1%, respectively, when trained on time-frequency features. These results show that both models effectively distinguish between ON, OFF, and SLEEP motor states with minimal misclassification, supporting their robustness across diverse feature sets and patient groups. To further analyze stability, we plotted the MLP training and validation F1-score curves over 200 epochs (Figure 7), which showed rapid convergence and consistently high performance, confirming the model’s reliability for motor state classification.

### 4.2 Different data and patient groups

To further assess the generalizability and robustness of the models, we compared classification performance across two key dimensions: data sources (wearable sensors vs. clinical diaries) and patient sample size (three vs. thirteen patients). In the comparison using data from three patients, all models achieved high F1-Score across both wearable sensor and clinical diary data. However, deep learning models such as MLP and CNN-LSTM performed slightly better on clinical diary data, with the MLP achieving a f1-score over 94% using time-frequency features, as shown in Figure 8a.

When scaling the analysis to thirteen patients using clinical diary data, model accuracy slightly declined, which is expected due to increased inter-patient variability. Although the number of patients was increased, the amount of data contributed by each individual was often limited, adding variability and sparsity to the training set. Nevertheless, time-frequency features continued to yield strong performance across both sample sizes, highlighting their robustness and scalability, as shown in Figure 8b.

### 4.3 Leave one day out evaluation

To assess temporal generalization across recording sessions, we performed a leave-one-day-out (LODO) evaluation. In this setup, each recording day was iteratively held out as the test set, while the model was trained on all remaining days across all patients.

Under this evaluation, the model achieved a mean macro- F1 score of 39.2%±16.6% , with performance ranging from 0.0% to 92.6% across folds. In contrast, the random train–test split yielded a substantially higher macro- F1 score of 93.1% .

This large performance gap indicates that random splitting overestimates model performance by allowing temporal leakage between training and test data. In contrast, the LODO evaluation provides a more realistic estimate of generalization to unseen recording sessions.

A closer examination of the results suggests that the observed variability is influenced by dataset characteristics. In particular, class imbalance across patients, especially the underrepresentation of the OFF state, and the limited number of recording days for several patients contribute to unstable performance across folds. Additionally, some test days contain only a small number of samples or lack certain classes entirely, further challenging model generalization.

## 5 Discussion

Our results show that neural signals extracted 24/7 in PD patients implanted with an aDBS system with sensing capability can be successfully applied to detect motor states, allowing a reliable classification across patients.

The dataset used here is, to our knowledge, the largest ever applied (more than 4,400 h of continuous recording in a 1-month time frame, with more than 260,000 time points) to motor state classification problems, thus highlighting the large potential brought in the research context by DBS devices with LFP sensing. The 1-min resolution together with the availability of a full spectrum every 10 min enabled the capture of neurophysiological dynamics informative of patients’ motor states. The availability of such signals will be crucial in the future years to expand our knowledge on deep brain pathophysiology and allow personalized precision neuromodulation therapies for PD and novel DBS indications.

This study presents a comprehensive evaluation of machine and deep learning methods for classifying PD motor states using multimodal features derived from neural recordings, wearable sensors, and clinical diaries. The results demonstrate that LFP time-frequency features provide the strongest discriminative power for distinguishing between *ON*, *OFF*, and *SLEEP* motor states, consistently outperforming time-only or frequency-only feature sets.

Across datasets and cohort sizes, time–frequency features consistently showed the strongest discriminative power for motor state classification. Among traditional machine learning approaches, KNN showed the most stable performance, while MLP emerged as the best-performing deep learning model.

An additional consideration concerns the selection of deep learning architectures and hyperparameters. In this study, we adopted commonly used configurations from the literature, balancing model complexity and generalization performance, rather than performing an extensive hyperparameter search. This choice was motivated by the limited prior evidence on optimal architectures for chronic LFP-based motor state classification and by the need to avoid overfitting given the dataset size and variability across patients.

Although model performance was monitored using validation data and learning curves indicated stable convergence, more systematic optimization strategies, such as grid search or Bayesian optimization, as well as the use of early stopping, could further improve performance. Future work will systematically investigate these approaches to better tailor model architectures to the specific characteristics of chronic neural recordings.

Performance remained comparable when extending the analysis to the full cohort, supporting the robustness and generalizability of the proposed framework. These findings confirm that time-frequency representations reliably capture PD motor state fluctuations, more effectively than any other feature type. This observation aligns with earlier studies that have emphasized the importance of beta-band oscillations and dynamic spectral features for detecting PD-related motor fluctuations (Kühn et al., 2006; Beudel and Brown, 2016; Hjorth, 1970). In particular, wavelet-based time-frequency methods allow the detection of brief and transient spectral changes, which are closely tied to fluctuations in motor symptoms (Hirschmann et al., 2011; Houston et al., 2019).

Our approach also extends previous work by systematically integrating multimodal data streams (wearable sensors and patient diaries) and evaluating models across both small and larger patient cohorts, using a substantially higher number of recording hours and labeled time points compared to most previous studies in the field. Unlike many studies that rely solely on static features (e.g., mean beta amplitude), our models leverage dimensionality-reduced representations and robust classifiers to handle inter-subject variability and real-world settings. While PCA enabled efficient handling of high-dimensional data, it reduces the direct interpretability of individual features, as each component represents a combination of multiple neurophysiological variables.

Alternative dimensionality reduction approaches, such as LASSO regularization or recursive feature elimination, could be explored in future work to preserve feature interpretability while maintaining classification performance.

Performance was slightly lower with wearable-sensor labels than with diary labels, even in the same patients. This does not indicate lower sensor reliability, but reflects differences in how motor states are defined and timed between the two methods, which can introduce label noise and reduce classification performance.

The agreement analysis between diary-based and wearable sensor–derived labels produced a Cohen’s kappa value of 0.22, indicating fair agreement between the two methods. To better understand this difference, agreement was also examined across specific state groupings. For the comparison between ON and OFF states, the kappa value was 0.19, indicating slight agreement. In contrast, agreement between SLEEP and non-SLEEP states was higher, with a kappa value of 0.23, which still falls within the range of fair agreement.

These results suggest that wearable sensors and clinical diaries are more consistent in identifying sleep-related states than in distinguishing motor ON and OFF conditions. This likely reflects fundamental differences between subjective patient-reported states and objective sensor-derived measurements, particularly for motor fluctuations, which are more difficult to capture using wearable signals. This mismatch introduces label uncertainty, particularly for ON/OFF classification, and may affect both model training and evaluation by introducing noise in the ground truth labels.

The ability of MLP and KNN to generalize across diverse patients using only time-frequency features suggests that future systems may rely less on labor-intensive manual labels (e.g., clinical diaries) while still achieving high F1-Score by using wearable sensors with the reliable biomarkers for motor-state prediction. By enabling robust motor state decoding in naturalistic conditions, these approaches can support the development of intelligent aDBS systems that dynamically adjust stimulation based on real-time neural and behavioral signals.

The clear difference between random split and leave-one-day-out performance shows the importance of temporally independent evaluation protocols. While random splits suggest near-perfect classification performance, they fail to capture the challenges associated with real-world deployment, where models must generalize to unseen recording sessions.

The observed performance degradation under leave-one-day-out evaluation can be largely attributed to characteristics of the dataset. First, class imbalance is pronounced, with the OFF state being significantly underrepresented or absent in some patients, making it difficult for the model to learn robust class boundaries. Second, several patients contribute only a limited number of recording days, restricting the model’s ability to capture stable temporal patterns and increasing susceptibility to overfitting to day-specific features. Third, variability in neural signals across days, combined with differences in sample size and class distribution per day, leads to substantial fluctuations in performance, with some days yielding high accuracy and others resulting in near-random predictions.

Together, these factors show the challenges of learning generalizable representations from limited and heterogeneous datasets collected over time.

To further investigate the effect of class imbalance, we compared several imbalance-handling strategies (Table 5). All approaches improved the detection of the minority OFF class compared to the unbalanced baseline. Random oversampling achieved the highest recall for the OFF class, but at the expense of reduced precision, suggesting an increased rate of false positives.

In contrast, the cost-sensitive approach provided a more balanced trade-off between precision and recall while maintaining stable overall performance, without introducing duplicated or synthetic samples. Synthetic data generation methods such as SMOTE and ADASYN yielded intermediate improvements but did not consistently outperform cost-sensitive learning.

These findings suggest that cost-sensitive learning represents a robust and reliable strategy for handling class imbalance, particularly given the clinical importance of avoiding false positive OFF detections. This is particularly important in clinical applications, where false positive OFF detections may lead to inappropriate stimulation adjustments.

From a system implementation perspective, an important consideration concerns how such decoding algorithms could be deployed within aDBS frameworks. While implantable neurostimulators offer the advantage of continuous sensing and closed-loop stimulation, they are constrained by limited computational resources, memory, and power consumption. As a result, executing the full feature extraction and classification pipeline entirely on the implantable device may not always be practical, particularly for time–frequency representations and deep learning models.

A more feasible approach may involve a hybrid architecture, in which lightweight feature extraction and state estimation are performed on-device for real-time stimulation control, while more computationally intensive model training, refinement, and personalization are carried out off-device, for example, using an external processing unit or cloud-based infrastructure. Such a strategy would allow models to be updated using larger datasets collected over time, including new days or additional patients, while maintaining low-latency and energy-efficient operation at the implant level.

In the present framework, motor state predictions are generated at a 10-min resolution, reflecting the feature extraction window and the way data are recorded by the implanted device. While this temporal resolution provides stable estimates of motor states, it may limit the detection of rapid state transitions. This limitation is mainly due to the data recording setup rather than the modeling approach itself. Future work will explore strategies to improve temporal responsiveness within these constraints, such as combining predictions across consecutive windows or integrating additional signals with higher temporal resolution.

In addition, prediction uncertainty is an important consideration for safe deployment in closed-loop systems. Practical implementations may use confidence-based strategies, such as probability thresholds, combining predictions across consecutive windows, or fallback stimulation policies when predictions are uncertain, to reduce the risk of inappropriate stimulation adjustments.

Overall, although the proposed framework demonstrates strong classification performance, it does not yet directly translate into immediate clinical applicability. It should be considered a preliminary step toward personalized adaptive DBS rather than a deployable clinical solution, as several practical challenges remain, including limited temporal resolution, computational constraints, and the impact of misclassifications.

Despite these limitations, this study shows that LFP-based features can reliably capture motor states across a large longitudinal dataset, representing an important step toward future adaptive DBS systems.

## 6 Limitations

Despite the encouraging results, several limitations should be noted. An important methodological consideration relates to the temporal resolution of clinical diary annotations. In this study, diary entries recorded at 30-min intervals were expanded to higher temporal resolution to match neural data. This approach assumes that motor states remain relatively stable within each 30-min interval; however, Parkinson’s disease symptoms may fluctuate over shorter time scales, particularly during medication transitions. As a result, this assumption may introduce some temporal uncertainty and potential label imprecision.

In addition, the wearable sensor dataset was limited to three patients, which reduces the generalizability of sensor-based findings. Although diary-based analyses included more patients, the number of samples per class was unbalanced, introducing variability in the dataset. Furthermore, agreement analysis showed that wearable sensors and clinical diaries were more consistent in detecting sleep-related states than motor ON/OFF states, highlighting the greater difficulty of accurately capturing motor fluctuations using sensor-based labeling. This discrepancy may introduce additional label uncertainty, particularly for ON/OFF classification.

In addition, periods of non-wear in the wearable sensor data were grouped under the SLEEP class, which may introduce misclassification, as inactivity due to non-wear does not necessarily correspond to true physiological sleep states.

Moreover, although the proposed framework achieved strong performance under the evaluated experimental settings, its generalization to unseen days remained limited. Using leave-one-day-out evaluation with time–frequency features and the MLP classifier, the macro F1-score is decreased. This performance drop shows the gap between random train–test evaluation and more realistic temporal generalization.

Notably, this performance is substantially lower than the 93.1% macro- F1 obtained using random train–test splitting, indicating that random splits may lead to overly optimistic estimates of model performance due to temporal leakage.

This reduced generalization performance is likely driven by several dataset characteristics, including class imbalance (particularly the underrepresentation of the OFF state), the limited number of recording days per patient, and variability in neural signals across days. These factors make it difficult for the model to learn stable patterns that generalize across time.

To estimate chance-level performance, we performed permutation testing (Ojala and Garriga, 2010) by randomly shuffling the test labels 100 times, yielding a mean macro- F1 score of 0.33±0.01 . The leave-one-day-out result therefore remained above chance level, indicating that the model still captures informative neural patterns despite reduced generalization.

Overall, the combination of limited temporal coverage and dataset imbalance suggests that the current dataset may not be sufficient to fully capture inter-day variability, which is critical for robust generalization in real-world settings.

To develop a more generalizable model capable of reliable performance on unseen days or new patients, future work should focus on increasing the number of participants and extending the duration of continuous recordings. A larger and more diverse dataset would better capture inter-day and inter-subject variability, improving robustness and supporting real-world deployment in aDBS systems.

## 7 Conclusion

In conclusion, this study demonstrates that long-term chronic recordings in ecologic settings can be successfully applied in advanced analysis frameworks combining multimodal data with time-frequency features and well-optimized models (KNN and MLP) to enable robust decoding of PD motor states. These insights contribute to improving aDBS systems in adapting current delivery dynamically to the patient’s needs and without requiring daily diaries. Future work will investigate this hybrid deployment paradigm and evaluate how model complexity and personalization strategies can be balanced to enable reliable and scalable aDBS systems in real-world clinical settings.

## Data Availability

The raw data supporting the conclusions of this article will be made available by the authors, without undue reservation.
